# Search for Expression Marker Genes That Reflect the Physiological Conditions of Blossom End Enlargement Occurrence in Cucumber

**DOI:** 10.3390/ijms25158317

**Published:** 2024-07-30

**Authors:** Rui Li, Runewa Atarashi, Agung Dian Kharisma, Nur Akbar Arofatullah, Yuki Tashiro, Junjira Satitmunnaithum, Sayuri Tanabata, Kenji Yamane, Tatsuo Sato

**Affiliations:** 1 United Graduate School of Agriculture, Tokyo University of Agriculture and Technology, 3-8-1, Saiwaicho, Fuchu 183-0054, Japan; lirui1212000@gmail.com (R.L.);; 2 Center for International Field Agriculture, Research and Education, College of Agriculture, Ibaraki University, Ami 4668-1, Ami, Inashiki 300-0331, Japansayuri.tanabata.i@vc.ibaraki.ac.jp (S.T.); 3 Faculty of Agriculture, Universitas Gadjah Mada, Jl. Flora No. 1 Bulaksumur, Yogyakarta 55281, Indonesia; agungbiotech@gmail.com (A.D.K.); akbar@ugm.ac.id (N.A.A.); 4 Organization for the Strategic Coordination of Research and Intellectual Properties, Meiji University, Kawasaki 214-8571, Japan; mpu_js@meiji.ac.jp; 5 School of Agriculture, Utsunomiya University, Mine 350, Utsunomiya 321-8505, Japan; yamane@cc.utsunomiya-u.ac.jp

**Keywords:** cytokinin, deformation, respiration rate, sugar starvation

## Abstract

Blossom end enlargement (BEE) is a postharvest deformation that may be related to the influx of photosynthetic assimilates before harvest. To elucidate the mechanism by which BEE occurs, expression marker genes that indicate the physiological condition of BEE-symptomatic fruit are necessary. First, we discovered that preharvest treatment with a synthetic cytokinin, N-(2-Chloro-4-pyridyl)-N’-phenylurea (CPPU), promoted fruit growth and suppressed BEE occurrence. This suggests that excessive assimilate influx is not a main cause of BEE occurrence. Subsequently, the expression levels of seven sugar-starvation marker genes, *CsSEF1*, *AS*, *CsFDI1*, *CsPID*, *CsFUL1*, *CsETR1*, and *CsERF1B*, were compared among symptomatic and asymptomatic fruits, combined with and without CPPU treatment. Only *CsSEF1* showed a higher expression level in asymptomatic fruits than in symptomatic fruits, regardless of CPPU treatment. This was then tested using fruits stored via the modified-atmosphere packaging technique, which resulted in a lower occurrence of BEE, and the asymptomatic fruits showed a higher *CsSEF1* expression level than symptomatic fruits, regardless of the packaging method. *CsSEF1* codes a CCCH-type zinc finger protein, and an increase in the expression of *CsSEF1* was correlated with a decrease in the fruit respiration rate. Thus, *CsSEF1* may be usable as a BEE expression marker gene.

## 1. Introduction

**Figure 1:**
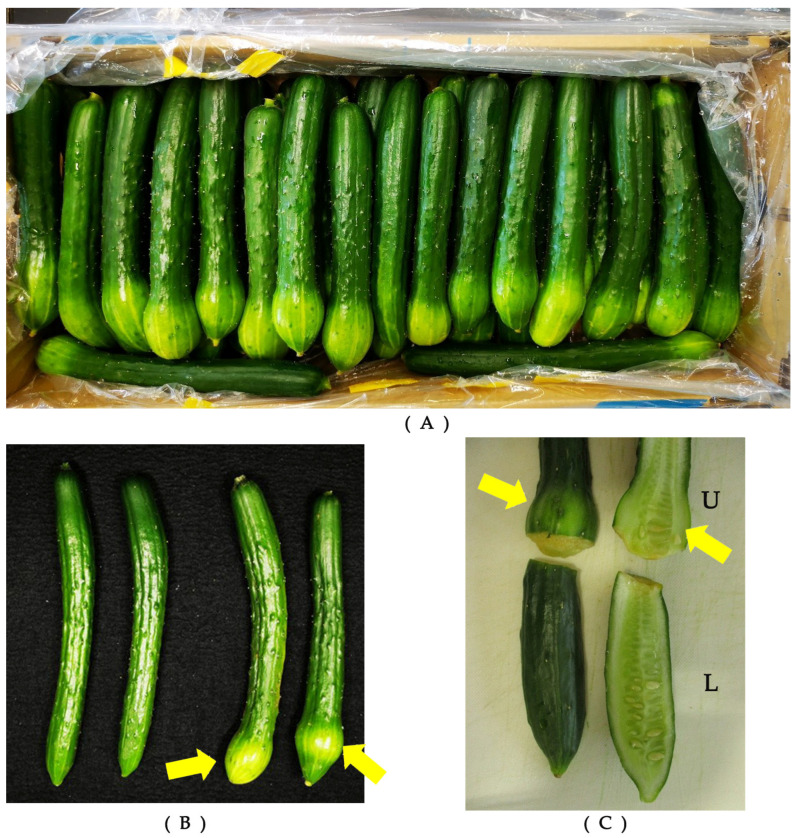
Symptom of blossom end enlargement. (**A**) Symptomatic fruits occurred during real distribution. (**B**) Left, asymptomatic fruits; right, symptomatic fruits. (**C**) Occurrence in cut fruits. Arrows indicate enlarged parts. U and L mean upper (peduncle side) and lower (blossom side).

Blossom end enlargement (BEE) [1] has been reported as a postharvest deformation under the tentative name of “apex enlargement” that occurs frequently in young cucumber fruits during summer outdoor cultivation [2,3,4]. The blossom ends of fruits can enlarge with etiolation when the package is opened at the store, although no signs are observed before shipment (Figure 1A,B). The enlarged portion invariably occurs on the blossom end side, even if divided into upper and lower halves (Figure 1C). This phenomenon decreases their quality and impacts cucumber sales, resulting in millions of dollars in losses in Japan annually.

The morphology of BEE is similar to the preharvest deformation of “bottle gourd-shaped fruit”, which occurs under conditions when plant vigor has weakened at the end of cultivation [5,6]. Elevated temperature during fruit growth has been identified as a factor leading to the occurrence of BEE, and respiratory suppression by Modified Atmosphere (MA) packaging [7,8] and low temperatures are effective in suppressing the occurrence of BEE [3,4]. BEE must be avoided in the commercial production of cucumber; therefore, pre-cooling using MA packaging films has been adopted in some production areas despite the high equipment and material costs.

N-phenyl-N-(2-chloro-4-pyridyl) urea (CPPU), as a cytokinin-type plant growth regulator [9], has been tested as a preharvest or postharvest treatment for fruits and vegetables. It has been reported that CPPU treatment increases the flesh firmness of cucumber at harvest [10] and that the cucumber fruit had the best fruit-setting ratio and subsequent development [11]. Satitmunnaithum et al. [1] discovered that the formation of BEE was related not only to the postharvest storage conditions but also to the preharvest cultivation environment, such as its temperature and solar irradiation, and plant conditions, such as the influx of photosynthetic assimilates as a result of the sink–source balance. Specifically, it has been proposed that one of the causes is a lack of plant hormone influx due to early harvest. However, the mechanism of BEE occurrence has not been proven unambiguously. BEE symptoms are similar to bottle gourd-type fruits; thus, it was suggested that both fruit deformations may have similar triggers [3]. To elucidate this mechanism, expression marker genes that indicate the physiological condition of BEE symptomatic fruit must be developed. Therefore, we examined seven genes related to the occurrence of “bottle gourd-type fruits” regarding fruit sugar starvation [6,12,13,14,15,16]. Cucumis sativus Somatic Embryogenesis Zinc Finger 1 (*CsSEF1*) was originally discovered as a gene that is upregulated during somatic embryogenesis [17], and this increase in the expression of *CsSEF1* is correlated with the decline in the fruit respiration rate [6]. The expression of *CsSEF1* is upregulated in malformed fruit induced by salinity stress in which the development of the placenta is arrested [13]. The transcript level of *CsSEF1* is lower under normal fruit development [12]. The expression of two other genes, the asparagine synthetase 1-like gene (*AS*) and the homolog of Cucumis sativus Fruit Defoliation Induced 1 (*CsFDI1*), has been previously reported as a control marker for sugar starvation in *Arabidopsis* [18,19]. The transcript levels of *CsFDI1* and *AS* are relatively high under normal fruit development [12]. Subsequently, other gene expression analyses were conducted using phytohormone-related genes to identify marker genes that reflect the physiological conditions of BEE symptomatic and asymptomatic fruits with and without CPPU. Plant hormones such as auxin stand out for their dominant function in morpho- and organo-genic processes, their formation of organs, and their regulation of tropic responses. Three auxin-related genes were also tested, namely FRUITFUL-like MADS-box gene (*CsFUL1*), which inhibits the expression of auxin transporters *PIN-FORMED1 (PIN1)* and *PIN7*, resulting in decreases in auxin accumulation in fruits [20] and mutation in the cucumber ortholog of *PINOID* (*CsPID*), a protein kinase that mediates polar auxin transport, resulting in widespread effects on cucumber plant development [21].

Furthermore, ethylene is a plant hormone that plays an important role in fruit ripening, plant growth, maturation, and signaling for self-defense mechanisms [22]. Here, the ethylene receptor-related genes *CsETR1* and *CsERF1B* were tested. A selected marker gene, *CsSEF1*, was tested for MA-packaged fruits as another preventive technique against BEE, and its effectiveness was confirmed. It is expected that a functional analysis of this gene will contribute to elucidating the mechanism by which BEE occurs.

## 2. Results

### 2.1. Effects of CPPU on Fruit Growth

**Figure 2:**
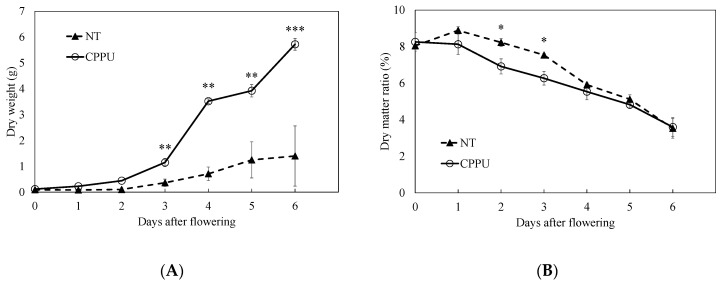
Effect of CPPU on fruit growth in cucumber. (**A**) Dry weight and (**B**) dry-matter ratio. NT: non-treatment. CPPU: CPPU application to the fruits at 100 mg L^−1^ at anthesis. *, **, and *** indicate significant differences between treatments at that time according to a *t*-test at *p* < 0.05, 0.01, and 0.001, respectively. Vertical bars are standard error bars (*n* = 7, R = 4).

A cotton swab was used to apply a non-dripping amount of the diluted CPPU onto the fruit surface’s peduncle end or bloom end treated (CPPU, hereafter). There was no difference in dry weight between the treated fruits and the untreated fruits (NT, hereafter) two days after flowering. The dry weight of CPPU-treated fruits started to increase more rapidly than that of NT fruits from three days after flowering, and the difference was four times as much at six days after flowering; here, the fruits treated with CPPU grew fastest on the third to the fourth days after flowering, increasing by 2.37 g, followed by those treated with CPPU on days five to six after flowering, increasing by 1.8 g (Figure 2A). The dry-matter ratio was approximately 8% at flowering, and it decreased to 4% six days after both treatments (Figure 2B). It was higher in the CPPU-treatment fruits than in the NT fruits two and three days after treatment.

### 2.2. Effects of CPPU on the Occurrence of BEE

**Figure 3:**
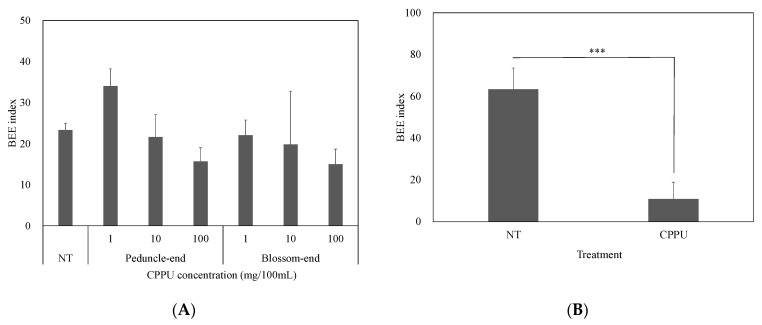
Effect of CPPU on the occurrence of BEE. (**A**) Postharvest treatment. There was no significant difference among treatments according to the Tukey–Kramer test; bars are error bars (*n* = 4–6). (**B**) Preharvest treatment at 100 mg L^−1^. NT: non-treatment. CPPU: CPPU application at anthesis. Vertical bars are standard error bars (*n* = 3–4), and *** indicates a significant difference between treatments according to *t*-test (*p* < 0.001).

In the postharvest treatment, there was no difference in the BEE index among the treatments after six days of storage. There was also no difference between the parts to which CPPU was applied in fruits (Figure 3A). In contrast, the BEE index in the preharvest CPPU treatment was lower than that in NT (Figure 3B).

### 2.3. Gene Expression of Sugar Starvation-Related Genes

**Figure 4:**
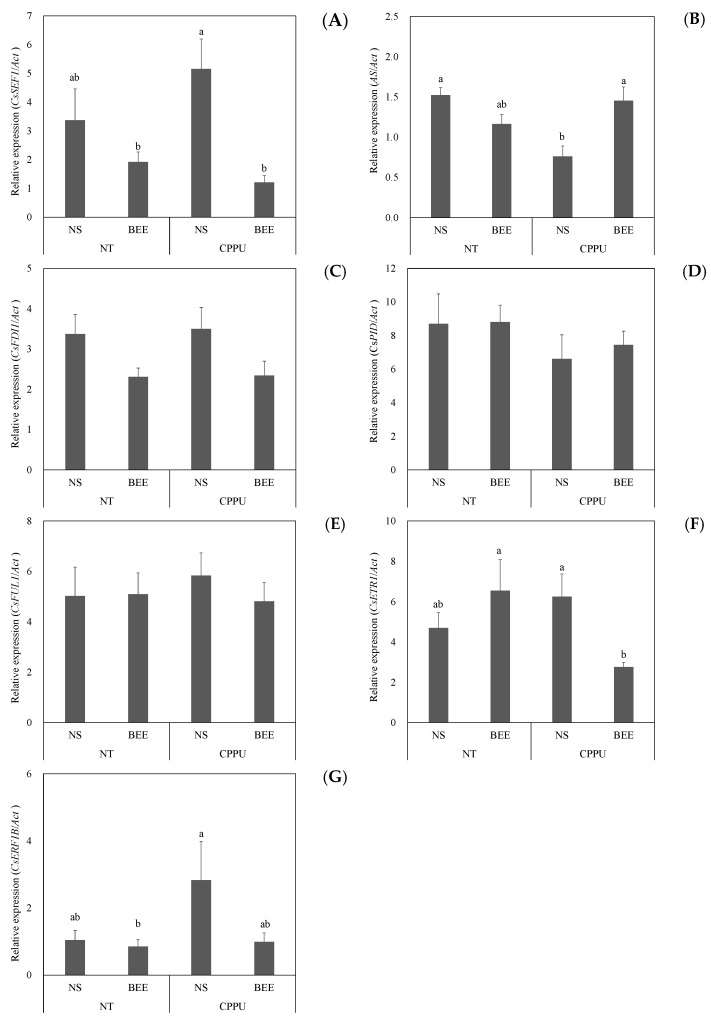
Expression of sugar starvation-related genes in cucumber fruits after six days of storage: (**A**) *CsSEF1*, (**B**) *AS*, (**C**) *CsFDI1*, (**D**) *CsPID*, (**E**) *CsFUL1*, (**F**) *CsETR1*, and (**G**) *CsERF1B*. CPPU: 100 mg L^−1^ of CPPU treatment was applied at anthesis. NT: non-treatment. RNA was extracted from the mesocarp of the blossom end of the fruits and subjected to quantitative PCR, and gene expression was normalized using actin expression. Vertical bars are standard error bars (*n* = 5–7). Different letters above the bars indicate significant differences (*p* < 0.05) determined using Tukey–Kramer’s test.

The relative expression levels of seven genes associated with similar deformation bottle gourd-type fruits were evaluated. The expression levels of *CsSEF1* were 3.37, 1.92, 5.16, and 1.21 in asymptomatic fruits without CPPU (NT-NS), symptomatic fruits without CPPU (NT-BEE), asymptomatic fruits with CPPU (CPPU-NS), and BEE-symptomatic fruits with CPPU (CPPU-BEE), respectively. The asymptomatic fruits, namely NT-NS and CPPU-NS, showed higher values than the symptomatic fruits, namely NT-BEE and CPPU-BEE (Figure 4A). In *AS*, the expression levels were 1.52, 1.16, 0.76, and 1.45 in NT-NS, NT-BEE, CPPU-NS, and CPPU-BEE, respectively, and the expression levels of CPPU-NS were lower than those of NT-NS, NT-BEE, and CPPU-BEE (Figure 4B). No differences were detected in *CsFDI1*, *CsPID*, and *CsFUL1* among treatments (Figure 4C–E). In *CsETR1*, expression levels were 4.70, 6.55, 6.26, and 2.76 in NT-NS, NT-BEE, CPPU-NS, and CPPU-BEE, respectively, and the expression levels of NT-BEE and CPPU-NS were higher than those of CPPU-BEE (Figure 4F). In *CsERF1*, expression levels were 1.04, 0.85, 2.83, and 0.99 in NT-NS, NT-BEE, CPPU-NS, and CPPU-BEE, respectively, and the expression level of CPPU-NS was higher than that of NT-BEE (Figure 4G).

### 2.4. BEE Occurrence and Expression of CsSEF1 in MA Packaging Film

**Figure 5:**
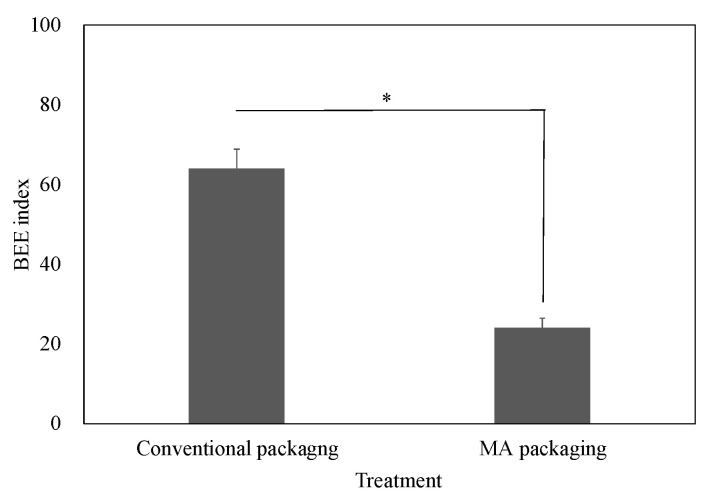
Effect of MA packaging film on the occurrence of BEE. MA packaging: fruits were stored within MA packaging film. Conventional packaging: fruits were stored within conventional packaging film composed of biaxially oriented polypropylene. Vertical bars are standard error bars (*n* = 6), and * indicates a significant difference between treatments according to *t*-test (*p* < 0.05).

**Figure 6:**
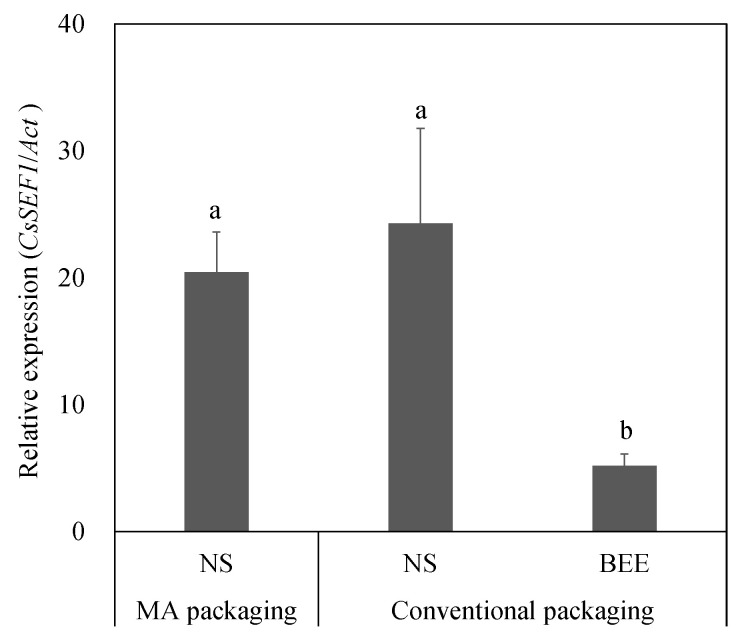
Sugar-starvation marker gene *CsSEF1* was expressed in cucumber fruits after six days of storage, using MA packaging film. MA packaging: fruits were stored within MA packaging film. Conventional packaging: fruits were stored within conventional packaging film composed of biaxially oriented polypropylene. RNA was extracted from the mesocarp of the blossom end of the fruits and subjected to qPCR, and gene expression was normalized using actin expression. Vertical bars are standard error bars (*n* = 6; Tukey’s test, *p* < 0.05). Different letters above the bars indicate significant differences (*p* < 5%) determined using Tukey–Kramer’s multiple-range test.

The occurrence of BEE was 2.7 times higher in conventional packaging than in MA packaging (Figure 5). Contrarily, the expression levels of *CsSEF1* were 3.9–4.6 times higher in asymptomatic fruits in both MA and conventional packaging, respectively, than in symptomatic fruits in conventional packaging (Figure 6).

## 3. Discussion

BEE is a postharvest deformation that poses a significant challenge in outdoor cucumber cultivation during the summer in Japan. Understanding the underlying mechanism of BEE and devising effective strategies to mitigate or delay cucumber deformation are important. To elucidate the mechanism by which BEE occurs, expression marker genes that indicate the physiological condition of BEE-symptomatic fruit are necessary. The functional analysis of this gene will elucidate the mechanism by which BEE occurs. Prior research has suggested that preharvest hormonal activity in fruits may be implicated in the BEE mechanism [1].

Consequently, we embarked on elucidating the effects of CPPU pretreatment on BEE, aiming to reduce BEE occurrence. Furthermore, post-harvest MA packaging of fruits has also been identified as a potential factor influencing the BEE mechanism. This method is employed to regulate storage conditions post-harvest, with the dual purpose of reducing BEE and exploring its mechanisms.

The preharvest application of CPPU reduced the degree of postharvest development of BEE, although the postharvest application of CPPU had no effect. These results suggest that the fate of fruit concerning whether or not it develops BEE is already determined before harvest, and cytokinin is associated with the trigger of BEE. Satitmunnaithum et al. [1] discovered that the fruits that grew in shorter days until harvest after flowering tended to experience a higher rate of BEE and suggested that if the fruit is harvested before a certain trigger substance can be fully synthesized in the fruit, the supply of that substance from outside of the fruit is cut off, leading to the onset of BEE. According to this theory, cytokinin may be the trigger substance. Morphologically, BEE is caused by localized swelling of the placenta [23], though cytokinin causes the entire fruit to swell through cell expansion [24].

During the period in which our field experiments were conducted, it took from five to eight days for cucumbers to develop, from flowering to harvest [1]. In the days between flowering and harvest, three possibilities were suggested: the need for cytokinins was lost, sensitivity to exogenous cytokinin was lost, or sufficient endogenous cytokinin was synthesized in the fruit. As shown in Figure 2, fruit growth was promoted by applying CPPU at the flowering stage. In BEE fruits, Nishizawa et al. [23] reported that the increase in the diameter of the swollen portion was due to placental swelling. On the other hand, cytokinins are thought to increase cucumbers’ whole fruit size and fresh weight through cell expansion [24].

The symptoms of BEE are similar to those of bottle gourd-type fruits; thus, both fruit deformations may have similar triggers [3]. Bottle gourd-type fruits tend to occur due to defoliation, root cutting, excessive soil moisture, potassium deficiency, and low soil temperature [5]. Maintaining the ground surface temperature and potassium application can effectively suppress this [25]. However, Satitmunnaithum et al. [1] reported that high temperatures, extended exposure to solar radiation, and a large leaf area concerning the number of fruits increase the occurrence of BEE, and fruits that reach harvest size shorter after flowering are more likely to develop BEE; therefore, BEE tends to occur under source-heavy conditions in the sink–source balance. Furthermore, our results also demonstrate that CPPU suppressed the occurrence of BEE, despite the promotion of the growth of cucumber being inconsistent with that in previous reports. It can be suggested that the results indicate that the fruit growth rate is not a direct trigger of BEE (Figure 1).

Igarashi et al. [4] reported no differences among the varieties in the occurrence of BEE. This experiment was conducted in a fully opened greenhouse, using a non-parthenocarpic variety, so all flowers were thought to be pollinated, even when CPPU was used. However, a previous study reported that BEE occurs mainly in fertilized fruits, but at a low frequency in parthenocarpic fruits [3,4]. It is likely that some trigger that causes placental expansion, regardless of whether a fertilized embryo is present, is related to the onset of BEE. Furthermore, Shishido et al. [26] reported a concentration gradient of cytokinins from the peduncle end to the blossom end based on the results of tracing a synthesized cytokinin benzyl adenine labeled with 14C. Kojima and Murata [27] reported that IAA is more concentrated at the bloom end than at the peduncle end in the pericarp and is transported more toward the peduncle from any part of the pericarp. In the placenta, IAA from the tissue near the peduncle is transported toward the peduncle end, and IAA from the tissue in the center, or the bloom end, is transported toward the bloom end. As shown in Figure 1C, BEE develops at the blossom end of excised fruits, similar to the polar transport of auxin. Cytokinin may suppress BEE by inhibiting polar transport in fruits.

The expression level of *CsSEF1* was lower in BEE fruits than in NS fruits, regardless of CPPU treatment (Figure 4A). MA packaging also suppressed BEE, and downregulation of *CsSEF1* was observed. Tazuke and Asayama [6] reported that the expression of *CsSEF1* was temporally correlated with the decline in the fruit respiration rate. The main effect of MA packaging film is to reduce the oxygen concentration by modifying the film’s permeability and to inhibit respiration by increasing the carbon dioxide concentration [28,29]. These results suggest that *CsSEF1* is available as a marker of BEE occurrence by reflecting the respiration rate.

Furthermore, cytokinin is thought to have two roles in inhibiting BEE. One is the respiratory inhibition effect as described in peas [30], asparagus [31], and oats [32], and the CPPU used in this study is thought to have suppressed the secondary growth of fruits after harvest through respiratory inhibition. The other is the regulation of the polar transport of auxin. CPPU probably prevented auxin from accumulating at the blossom end. In this study, the effect of CPPU on the expression level of *CsPID* [33] and *CsFUL1* [20], which is involved in auxin polar transport, was unclear. The possibility that CPPU may regulate the expression of other homologs or be involved in further downstream reactions remains to be examined.

The occurrence of BEE continues to be suppressed, even after opening the MA package [3]. This suggests a limited period in which fruits react to the trigger, and once the BEE trigger is canceled, it will not turn on again. To clarify the triggering factors, combined with the results of CPPU, we speculate that its triggering may involve auxin transport. Therefore, we chose some genes related to plant hormones among the seven candidate genes for their association with bottle-shaped fruit. *CsETR1* and *CsERF1B* are related to the ethylene receptor [34] and ethylene response [35]. Although the expression levels of these genes differed among the treatments, no relationship with BEE was detected. This suggests that they may not directly influence the occurrence of BEE. The gene functions of *CsFDI1* and *CsSEF1* have not been clarified, despite being tested as sugar-starvation markers [6,12,13,14,15,16].

Our study identified the marker genes associated with BEE in cucumbers and discovered an effective method to prevent this deformation in the potential application of CPPU pretreatment and post-harvest MA packaging to control BEE. These findings hold significant promise for improving the economic value of cucumbers by reducing postharvest losses. By understanding the role of these target genes and the hormonal mechanisms involved, we can develop targeted strategies to mitigate deformation, ensuring better quality and higher market value for cucumbers. However, the limit of our study is that their effectiveness across different cucumber varieties and environmental conditions needs further validation. Additionally, we need to explore alternative hormonal treatments to achieve consistent results. In the future, it will be necessary to expand the scope of investigation beyond sugar-starvation genes to comprehensive transcriptome-, enzyme-, and phytohormone-level analyses.

## 4. Materials and Methods

### 4.1. Plant Materials

This study was conducted between 2020 and 2023. Cucumbers (*Cucumis sativus* L. ‘Taibo I’) were cultivated as described by Satitmunnaithum et al. [1]; however, in 2023, the cucumber plants were grown hydroponically in fully opened greenhouses. Cucumber seedlings were grafted on the rootstocks ‘Tokiwa power Z2′ (*Cucurbita moschata* DUCH). The primary stem and first branches were pinched at the fifteenth and first nodes, respectively, while the remaining branches were undisturbed. Fruits estimated to weigh 80 g or more by visual inspection were harvested between 06:00 and 08:00 a.m. Each fruit was weighed, and 80–100 g fine-shaped fruits were selected for the experiment. Sowing and transplantation were performed on 30 April and 25 May in 2020, 30 April and 2 June in 2021, 22 April and 20 May in 2022, and 6 April and 10 May in 2023.

### 4.2. Effect of Postharvest CPPU Application on BEE Occurrence

In 2020, a commercial CPPU preparation (Fulmet liquid, Sumitomo Chemical Co. Ltd., Tokyo, Japan) was applied at three concentrations of 1, 10, and 100 mg L^−1^. NT treatment was compared as a negative control. Immediately after application, the fruits were packed into a plastic bag composed of biaxially oriented polypropylene film (300 × 150 × 0.025 mm^3^, New Bodon #25; Daiko Corporation, Miyagi, Sendai, Japan), with four air holes (diameter: 5.5 mm) on either side. Packages of fruits were stored for six days at 27 °C, under dark conditions, to observe the occurrence of BEE. The following BEE scores were given to each fruit based on visual observations six days after storage: 0 = normal fruit, 1 = slight enlargement, 2 = apparent enlargement, and 3 = extreme enlargement. The degree of BEE was evaluated using the BEE index, calculated using the following formula:

BEE index = (Σ(n × v)/N × Z) × 100(1)

where n is the severity score of the fruit, v is the number of samples, N is the highest score, and Z is the total number of samples [1]. Four-to-six fruits were subjected to each treatment, and the tests were repeated three times.

### 4.3. Effect of Preharvest CPPU Application on BEE Occurrence

Flowers that bloomed from 16 July to 10 August, from 10 June to 13 August, and from 2 June to 5 July were subjected to CPPU application in 2021, 2022, and 2023, respectively. Approximately 20 μL of 100 mg L^−1^ CPPU solution was applied to the peduncle of flowers immediately after flowering every morning, using a small brush. The non-treated fruits were used as negative controls. Four-to-seven fruits per treatment were placed in a packing bag, and the BEE index was evaluated for each bag after six days. The experiment was repeated three times.

### 4.4. Effect of CPPU on Fruit Development

In 2023, the growth processes of cucumber fruits were compared between CPPU and NT. The protocol for CPPU treatment was the same as described above to determine the effect of preharvest CPPU application on BEE occurrence. The fruit dry weight was measured every day until six days after flowering, and the dry-matter ratio was calculated. Seven fruits were used each time, with four replicates per treatment.

### 4.5. Effect of MA Packaging Film

Storage tests were conducted using an MA packaging film (FH film; Sumikasekisui Film Co., Ltd., Tokyo, Japan) from 10 June to 17 July 2023. The film was fashioned into a bag the same size as the conventional biaxially oriented polypropylene film bag described in Section 4.2. The same storage conditions were used to compare BEE occurrence between the fruits and conventional films. Four-to-six fruits were used for each treatment. The experiment was repeated thrice.

### 4.6. Gene Expression Analysis

To select the marker genes of BEE occurrence, fruits were classified into four types after six days of storage: NT-NS, NT-BEE, CPPU-NS, and CPPU-BEE, respectively. Here, only fruits that have a severity score of 3 (highest) were selected as samples for symptomatic fruits. Approximately 50 mg of mesocarp was cut from the blossom end, and RNA was extracted following the AGPC method (RNAiso Plus, Takara Bio, Shiga, Japan) and reverse-transcribed (Prime Script RT Master Mix, Takara Bio, Shiga, Japan) according to the manufacturer’s instructions. The quantitative real-time polymerase chain reaction (qPCR) was used to assess the relative expression levels. The evaluation was conducted using the obtained DNA template with the KOD SYBR qPCR mix (Toyobo, Osaka, Japan) and the CFX Connect Real-Time PCR system (Bio-Rad, Hercules, CA, USA). The seven target genes consisted of sugar-starvation marker genes *CsSEF1*, *AS*, and *CsFDI1* [15]; auxin-related genes *CsPID* [33] and *CsFUL1* [20]; and ethylene-related genes *CsETR1* and *CsERF1B*, as shown in Table 1. And the melting curve of qPCR to certify specific amplification is shown in Supplementary Figure S1. Gene expression was normalized to that of actin. The cycling condition of qPCR was as follows: initial denaturation at 98 °C for 120 s, denaturation at 98 °C for 10 s, annealing at 60 °C for 15 s, and extension at 68 °C for 30 s up to 40 cycles. Furthermore, the expression level of *CsSEF1* was compared among symptomatic and asymptomatic fruits stored for six days in conventional packaging and asymptomatic fruits in MA packaging. Symptomatic fruits were not placed in MA packaging because there was only a severity score of 1 in this treatment. Six cucumber fruits of each type were used in the experiment, and the experiments were repeated three times to confirm reproducibility.

### 4.7. Statistical Analysis

The *t*-test was used for pairwise comparisons, and the Tukey–Kramer test was used for multiple comparisons. The EZR software (Easy R ver. 4.0.3; EZR ver. 1.54) [36] was employed.

## 5. Conclusions

According to the results of the preharvest CPPU treatment and storage test using MA packaging, a putative CCCH-type zinc finger protein, *CsSEF1*, was suggested to be available as an expression marker gene of BEE. This gene may reflect the respiration rate of the fruits.

## Figures and Tables

**Table 1:** Target genes in gene expression analysis.

Abbreviation in GenBank	Description in GenBank	Primer Sequence
*CsActa* AB698859.1	*Cucumis sativus* mRNA for actin, cultivar: Shimoshirazu jibai.	F 5’-TCTGTCCCTCTACGCTAGTGGAC-3’ R 5’-TCCAAACGGAGAATGGCATGAGG-3’
*CsSEF1* AJ870303.1	*Cucumis sativus* mRNA for putative CCCH-type zinc finger protein	F 5’-AGACCGATACCGGACTCAAC-3’ R 5’-TGTGAGCGAAGAAACAGACC-3’
*AS* XM_004143115.3	PREDICTED: *Cucumis sativus* asparagine synthetase	F 5’-TGAGGGTTCACCAGATTTGA-3’ R 5’-AATGGCATCAATCCCATCTT-3’
*CsFDI1* XM_031888327.1	PREDICTED: *Cucumis sativus* BTB/POZ domain-containing protein At1g63850-like, transcript variant X1	F 5’-AATGCCCTCTTCAGCACTCT-3’ R 5’-GAGGAAATGTTGCAGGGATT-3’
*CsPID* XM_031882167.1	PREDICTED: *Cucumis sativus* protein PIN-LIKES 7-like	F 5’-TCCATCGGCATTATGACTGAGC-3’ R 5’-ATCCACATGAAGAGTGTATACC-3’
*CsFUL1* XM_011652029.2	PREDICTED: *Cucumis sativus* truncated transcription factor CAULIFLOWER A, transcript variant X2	F 5’-GGCAAAAAGATAAAGGAGAAGGAG-3’ R 5’-ATGCTAAGAGATTGAAATGGCTGA-3’
*CsETR1* NM_001280633.1	*Cucumis sativus* ethylene receptor 1-like	F 5’-AACTCTTGTGGCAGTGGTCT-3’ R 5’-GTAGCCGTGCATCCCTTTCC-3’
*CsERF1B* XM_004134020.3	PREDICTED: *Cucumis sativus* ethylene-response factor C3	F 5’-TGCATTCATCTCCCCGCTTT-3’ R 5’-GGTGAAGGAGGATTCGGGTG-3’

## Data Availability

Data are contained within the article.

## Supplementary Materials

The following supporting information can be downloaded at https://www.mdpi.com/article/10.3390/ijms25158317/s1.
