# Supplementary material for: Search for Expression Marker Genes That Reflect the Physiological Conditions of Blossom End Enlargement Occurrence in Cucumber
Source: Int J Mol Sci. 2024 Jul 30;25(15):8317. doi: 10.3390/ijms25158317 (PMC11312178; doi:10.3390/ijms25158317)
Supplement: Supplementary file 1 [file ijms-25-08317-s001.zip › ijms-3089065-supplementary.pdf]

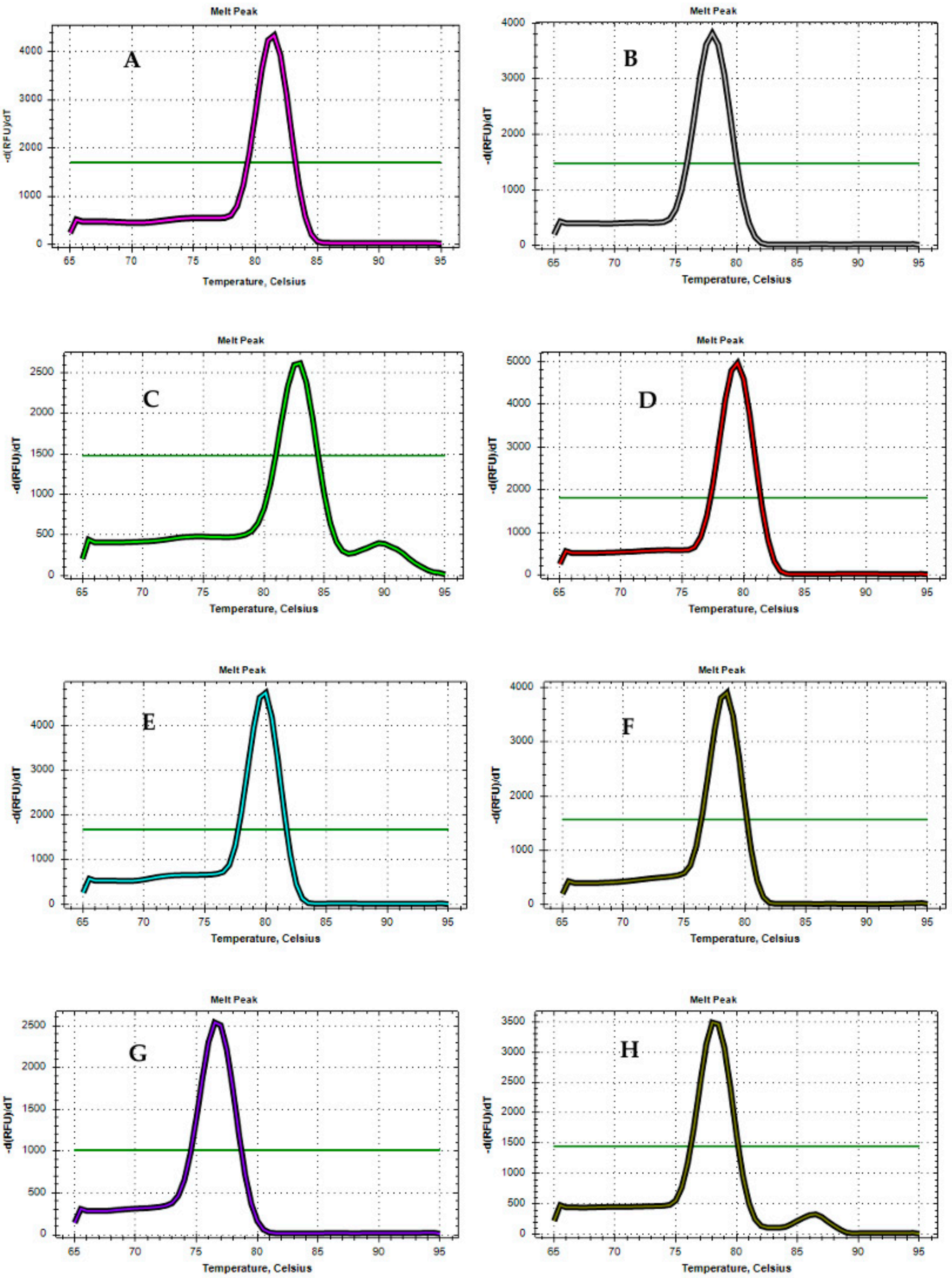

Figure S1: The qPCR melting curve of sugar starvation-related genes in cucumber fruits after six days of storage（A:*CsAct a*； B:*AS*； C:*CsSEF1*； D:*CsFDI1*； E:*CsPID*； F： *CsFUL1*； G: *CsETR1*； H: *CsERF1B*).The melting curve analysis was performed to verify the specificity of the qPCR amplification products, ensuring the absence of non-specific products and primer dimers. A sample was randomly selected for each primer.
